# Compare and Contrast of the Cellular Actions of Related Flavonoids, Apigenin and Chrysin

**DOI:** 10.3390/nu16234195

**Published:** 2024-12-04

**Authors:** Patrick Keefe, Prasanth Puthanveetil

**Affiliations:** 1 Chicago College of Osteopathic Medicine, Midwestern University, Downers Grove, IL 60515, USA; patrick.keefe@midwestern.edu; 2 Department of Pharmacology, College of Graduate Studies, Midwestern University, Downers Grove, IL 60515, USA

**Keywords:** flavonoids, apigenin, chrysin, cholesterol biosynthesis, transcriptomic analysis, metabolomics, uric acid

## Abstract

In this review, we provide an evidence-based approach to determine the cellular and systemic actions of two structurally similar flavonoids, apigenin and chrysin. We have clearly evaluated and charted the overlapping and diverging properties of these two sister flavonoids. Based on two separate Omics-based approaches by our group and independent reports from others, the cholesterol-lowering properties have been revealed. In addition, the prevention of uric acid biosynthesis and enhancement of ketogenesis have also been quite evident in these two flavonoids. Along with these overlapping functions, apigenin and chrysin have also demonstrated unique properties that allow them to stand out from each other. Chrysin has demonstrated abilities like downregulating alanine metabolism and pyrimidine synthesis, which could be helpful in metabolic diseases like cancer. In contrast, apigenin has demonstrated anti-oxidant and anti-inflammatory properties by enhancing endogenous anti-inflammatory lipids and upregulating vasoprotective metabolites, which could be beneficial for cardiovascular, renal, and cerebrovascular complications. Further validation studies using in vivo and translational approaches could provide us with better clarity regarding the use of these agents therapeutically and to treat a combination or pool of metabolic diseases.

## 1. Introduction

Phytochemicals are plant-based bioactive compounds whose isolation has long provided opportunities for naturally occurring pharmaceutical interventions. Roughly one-third of all novel drugs approved by the Food and Drug Administration [1,2,3] are rooted in naturally occurring phytochemicals [4]. Chrysin and apigenin are two such examples. They are structurally related polyphenols within a class of phytochemicals called flavonoids, the largest group of polyphenols [2,3]. They differ in structure solely by the addition of a 4′ hydroxyl group to apigenin’s phenol, which is itself attached to the second carbon of the chromone structure [2,3]. The chemical names for apigenin include 5,7,4′-Trihydroxyflavone and 5,7-dihydroxy-2-(4-hydroxyphenyl)chromen-4-one (IUPAC) [2,3]. Chrysin can also be referred to as 5,7-Dihydroxyflavone or 5,7-dihydroxy-2-phenylchromen-4-one (IUPAC) [2,3].

Apigenin is mostly found in food sources like chamomile tea, celery, parsley, artichokes and oregano [5]. Chrysin is found mostly in food sources like green vegetables, passion flowers, and honey [6].

There is an extremely wide range of methods, qualities, and forms that apigenin or chrysin is utilized in current studies, such that comparison and review are becoming challenging. Understanding the specific effects of these phytochemicals is paramount if they are to be considered within the clinical setting and for future work that aims to capture their effects in a targeted manner.

Here, we offer a perspective review that summarizes the specific activities mediated by chrysin and apigenin, both individually and combined. This summary follows past efforts by our lab [1,2], which articulated actions of chrysin and apigenin through both transcriptome analysis and measurement of widespread metabolomics. Understanding the specific cellular changes in metabolites and transcription factors offers an invaluable opportunity to understand chrysin and apigenin’s biological activity and pharmacological properties. Table 1 offers a summary of the crucial findings presented in our past omics-based works.

### Overlapping Effects of Both Chrysin and Apigenin

When considering biochemical pathways, Acetyl-CoA is a central molecule between many of these pathways. It provides a helpful reference point to assess changes in transcripts and metabolites related to those pathways that pass through it. By doing so, strong conclusions can be drawn about activities within the cell that capture its current state. Under normal circumstances, Acetyl-CoA is predominantly channeled into the TCA cycle. In well-fed states, when energy is in surplus, Acetyl-CoA is shunted to biosynthesis pathways. Typically, of high energy molecules such as Fatty Acids and Cholesterol for later use.

In our previous works [1,2], the primary pathway affected by both Chrysin and Apigenin was that of cholesterol biosynthesis. Transcriptome analysis demonstrated a statistically significant reduction in virtually all enzymes related to this pathway, including transcripts of the gene coding for the enzyme HMG-CoA Reductase. This, coupled with changing levels of various metabolites and transcripts discussed within this review, allows us to conclude that under the effects of both chrysin and apigenin, there is an apparent divergence of Acetyl-CoA towards ketogenesis and away from cholesterol synthesis and Fatty acid synthesis. 

## 2. Effects on Cholesterol Biosynthesis

**Figure 1:**
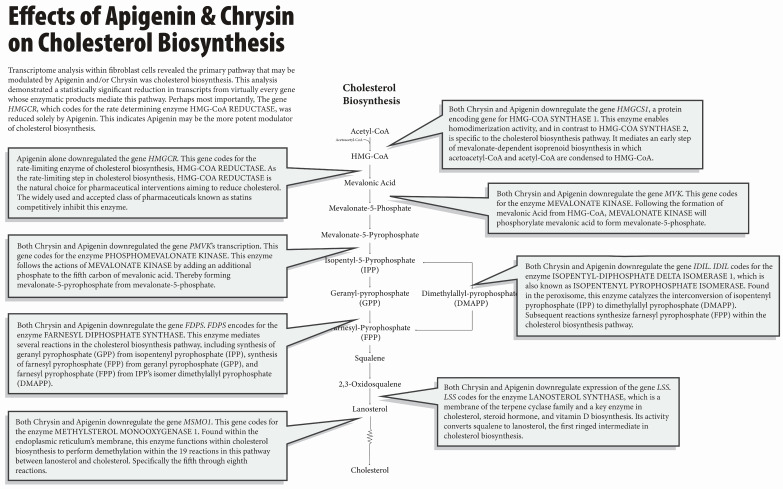
Regulation of cholesterol biosynthesis by apigenin and chrysin: This figure depicts how apigenin and chrysin regulated the enzymes involved in the mevalonate pathway or cholesterol biosynthesis pathway. The ability to downregulate multiple targets in the cholesterol biosynthesis pathway by apigenin and chrysin makes them unique candidates for therapeutics targeting hyperlipidemia or hypercholesterolemia.

The most prominent pathway that may be affected by apigenin and chrysin in our work is in cholesterol biosynthesis [3]. Major enzymes in the mevalonate pathway are regulated via sterol regulatory element binding proteins (Srebp1/2), which, under normal circumstances, are released from the endoplasmic reticulum when cellular cholesterol is low and act to upregulate cholesterol biosynthesis by binding to the sterol binding proteins responsive element (SRE) [7,8,9]. These genes will ultimately code for the enzymes involved in cholesterol biosynthesis. Secondarily, the low-density lipoprotein receptor (LDLR), which allows the delivery of cholesterol to the cell, is also a major Srebp target [7]. Figure 1 offers a summary of this process. It is natural to see how these two activities would correlate and interplay to maintain desired cholesterol levels.

**Figure 2:**
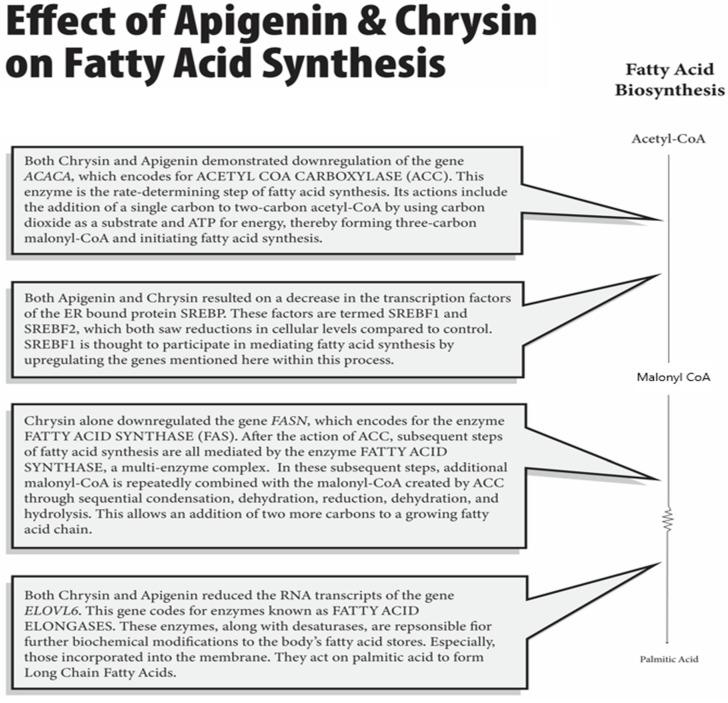
Regulation of fatty acid biosynthesis by apigenin and chrysin: The provided diagram clearly explains how apigenin and chrysin downregulate different targets that are involved in lipogenesis, decreasing cellular fatty acid levels.

When exposed to apigenin or chrysin, two transcriptional factors of this process, Srebf1 and srebf2, were both downregulated when exposed to either flavonoid. While the exact interplay and tissue-specific activities of these two factors remain elusive, Srebf2 is generally considered to correlate towards the upregulation of cholesterol biosynthesis and the LDLR and Srebf1 towards upregulating aspects of fatty acid synthesis [7,8,9]. Further, a decrease in the levels of LDLR was observed within the transcriptome analysis in our past work [1,2], which also indicates a reduction in the transcriptional and downstream effects of Srebp [7,8,9]. Such transcriptional changes correlate to the halting of cholesterol biosynthesis. Even more telling, downregulations of many transcriptional factors from genes coding for the enzymes of virtually every step of cholesterol biosynthesis were observed. Figure 2 displays the specific steps and transcript regulation within the cholesterol biosynthesis pathway that were downregulated by chrysin and/or apigenin [3], as shown in Figure 1. In addition, our past work analyzing the cell-wide metabolomics of mouse embryonic fibroblast cells demonstrated a reduction in the metabolites of 7-dehydrocholesterol and xanthosine [2]. Both these metabolites are intermediates of cholesterol and uric acid [2]. Their reduction indicates both these pathways are downregulated by the flavonoids Chrysin and Apigenin. 

Previous studies have also demonstrated the cholesterol-lowering effects of apigenin [10,11]. Our findings were also reflected in a 2019 work by Lu et al. that also demonstrated a reduction in the SREBP family of transcriptional factors [12]. Further, Zhang and Song et al., in a 2017 in vivo study, used apigenin in mice as a mechanism of treatment for lipidemia [13]. Their work demonstrated a dose-dependent reduction in total cholesterol and LDL in mice treated with apigenin compared to control, as well as reductions in transcriptional levels of mRNA related to HMG-CoA Reductase and LDLR [13]. 

## 3. Effects on Fatty Acid Synthesis 

As mentioned, fatty acid [14] synthesis is an alternate fate of Acetyl-CoA in the well-fed state. FAs are stored in lipid droplets within adipose tissue, which can later be catabolized via mitochondrial beta-oxidation for their high-energy potential. The rate-determining step of fatty acid synthesis is by the enzyme Acetyl-CoA Carboxylase (ACC). In our work [1,2], both chrysin and Apigenin demonstrated statistically significant downregulation of transcripts of the gene Acaca, which encodes for Acetyl-CoA Carboxylase, as depicted in Figure 2. This enzyme is the rate-determining step of fatty acid synthesis [15,16]. Its actions include the addition of a single carbon to two-carbon Acetyl-CoA by using carbon dioxide as a substrate and ATP for energy, thereby forming three-carbon malonyl-CoA and initiating fatty acid synthesis. Subsequent steps of the process are all performed by the enzyme Fatty Acid Synthase [15,16]. In these subsequent steps, additional malonyl-CoA is repeatedly combined with the malonyl-CoA created by ACC through sequential condensation, dehydration, reduction, dehydration, and hydrolysis [15,16]. This allows the addition of two more carbons to a growing fatty acid chain. Interestingly, chrysin alone downregulated the gene Fasn to significant levels, which encodes for the enzyme Fatty Acid Synthase [3], as shown in Figure 2.

Gomez-Zorita concluded similar findings to our own with an observed reduction in the transcriptional activity of enzymes of fatty acid synthesis, such as ACC and FAS, under the effects of Apigenin [17]. They also demonstrated an upregulation of molecules for lipolysis, such as ATGL and Perilipin by apigenin, and no increases in adipogenesis. Bumke-Vogt et al. similarly concluded the reduction in gluconeogenesis and lipogenesis through the enzymes ACC and FAS when cells were exposed to Apigenin [18]. Lu et al.’s 2019 work demonstrated that apigenin may influence the enzyme Fatty Acid Synthase. In that study, not only reductions in the Srebp transcriptional path leading to cholesterol synthesis, as described above, were observed, but there was a similar reduction in FAS via its Srebp1 transcript [12]. Iwase et al. also assessed the functioning of chrysin on SREBP and its transcriptional effects in human hepatoma Huh-7 cells [19]. Their work demonstrated a reduction in the transcriptional activity of the enzymes of fatty acid synthesis, such as FAS [19]. The group also explored the activities of the ubiquitin-proteosome degradation mechanism, which were found to be unaffected by Chrysin [19]. With these facts, they concluded that the reduction in mature SREBP is indeed due to reduced transcriptional activity and not from normal protein degradation.

Other past studies have additionally presented conclusions on how Apigenin and Chrysin may activate AMPK [12,19,20]. A molecule that phosphorylates and inactivates many enzymes, including those of fatty acid and cholesterol biosynthesis. Namely, Acetyl-CoA Carboxylase, Fatty Acid Synthase, and HMG-CoA-reductase [12,19,20]. Similarly, Zhou et al. in 2021 explored the increases in AMPK under the effects of Chrysin using various mice and cell models [20,21]. This, coupled with a reversal of the declining PI3K/Akt pathway and downregulation of the gluconeogenic enzymes, led to the conclusion that chrysin can reduce fatty acid synthesis [21]. Other works related to apigenin concluded it downregulates the expression of CD36, the receptor that allows for intestinal absorption of fatty acids; inactivation of PPARy, whose expression depends on CD36 and promotes adipogenesis; inactivation of lipolytic enzymes like ATGL, LPL, and DGAT2; and inhibition of pancreatic lipase, an enzyme that breaks down fatty acids for absorption [22,23,24,25,26,27].

## 4. Diversion of Acetyl-CoA to Ketogenesis

As stated previously, there exists an apparent diversion of the molecule Acetyl-CoA to Ketogenesis within cells exposed to chrysin and/or apigenin. That is, the formation of 3-hydroxybutyrate via an acetoacetate intermediate [3], as shown in Figure 2. When not in an aberrant state, this mechanism allows the body to transport these molecules, known as ketones, from the liver to other tissues, where they can be reformed into Acetyl-CoA and used for energy-creating processes locally [28]. It is readily understood that Acetyl-CoA acts as a nexus for several biochemical pathways, including the TCA cycle and subsequent electron transport chain [28], cholesterol synthesis, and fatty acid synthesis. Indirectly, Acetyl-CoA affects gluconeogenesis through allosteric downregulation of the Pyruvate Dehydrogenase Complex [29]. This Complex’s E1 subunit would perform decarboxylation of Pyruvate to form Acetyl-CoA and continue through aerobic respiration. When allosterically affected by levels of Acetyl-CoA (and others), pyruvate is rather reversed into the gluconeogenesis pathway [29].

As described, Chrysin and Apigenin were both observed to reduce measurable levels of target genes responsible for several enzymes in the fatty acid synthesis (Figure 2) and cholesterol synthesis pathways [3], shown in Figure 1. Cellular-wide metabolomics further elucidates the effect on the biochemical pathways of these two flavonoids. Such assessment demonstrated an upregulation of the enzyme 3-hydroxybutyrate Dehydrogenase 1, an oxidoreductase enzyme encoded by the gene Bdh1 by both chrysin and apigenin-affected cells [3]. This enzyme is responsible for the interconversion of 3-hydroxybutyrate and acetoacetate, an important step in ketogenesis. In addition, the transcript Slc16a6, Solute Carrier Family 16 member 6, was observed to be upregulated. This transcript encodes for the monocarboxylate transporter 6 (MCT6/7), a transporter that is involved in the influx and efflux of monocarboxylate molecules. Monocarboxylates include molecules that have just one carboxylate group, including lactate, pyruvate, and ketones. In our study of Chrysin and Apigenin, the transcript Slc16A6 was the fifth most upregulated transcript by both chrysin and apigenin [3]. Overall, the upregulation of a primary ketogenesis enzyme and a transporter for molecules involved in its process indicates ketogenesis may be induced by chrysin and apigenin, as shown in Figure 2. A search of the EBSCO database using the keywords “apigenin OR chrysin” and “Ketogenesis” revealed no search results besides our own works. This may indicate a need to further explore the correlation between these two molecules and the induction of ketogenesis.

## 5. Effects on Uric Acid Synthesis

Uric acid in excess is not only an issue with gout but also a trigger factor for many cardiovascular complications, including heart failure, vascular disease, and arrhythmia [30,31,32]. Hyperuricemia could be due to excess uric acid accumulation or underexcretion [33,34,35]. For example, hypertension itself can cause hyperuricemia from reduced blood flow through the kidney’s glomerulus, where uric acid would typically be filtered [36,37,38,39]. Conversely, proliferative disorders like cancers or psoriasis typically cause hyperuricemia from the high turnover of cells [40,41,42,43,44]. This results in a subsequent excess catabolism of the cell’s purines and an overproduction of uric acid [40,41,42,43,44]. The drug colchicine, which acts to inhibit microtubule formation and thus slow the turnover of cells, has long been a first-choice pharmacologic treatment [45]. Its described use for gout goes back decades if not centuries [45]. Today, it is more judiciously used due to severe side effects and the high non-compliance from those side effects. Yet, these newer options are highly specific for uric acid-lowering mechanisms. Based on randomized-controlled trials, cardiovascular health benefits have been reported for colchicine for patients suffering from pericarditis and coronary artery disease [1,45,46,47,48,49,50,51]. Liu et al., in 2023, assessed apigenin’s ability to lower uric acid levels [52]. The study also compared allopurinol to that of apigenin [52]. While both lowered uric acid levels, only apigenin showed improved blood urea nitrogen (BUN) and creatinine, which are markers or measures of kidney function [52]. Further, high-dose apigenin had a statistically significant greater uric acid lowering effect than allopurinol, a clinically approved uric acid lowering agent [52]. Li et al., in 2021, also demonstrated the ability of apigenin to lower uric acid levels. In their study, it was concluded that apigenin inhibits the renal transporter URAT1 and others, which allows for the reabsorption of uric acid. Causing the kidney to excrete uric acid to a greater extent [53]. Gan et al., in 2023, confirmed the uric acid-lowering effects of apigenin [54]. In their study, they developed a mechanism for assessing the inhibitory abilities of various flavones on the xanthine oxidase enzyme rather than focusing on serum uric acid levels [53,54]. Of the more than 30 compounds, Apigenin was ranked third highest in its dose-dependent inhibitory abilities on this enzyme. Uric acid lowering effects are also exhibited by chrysin. In 2021, Chang et al. reported the uric acid-lowering effect of chrysin in a rat model [55]. These works validate the hypouricemic property of these two similar flavonoids.

Changes in metabolomic measurement of the metabolites 7-dehydrocholesterol and xanthosine were observed when mouse embryonic fibroblast cells were exposed to either chrysin or apigenin [2]. These metabolites are involved in both the production of uric acid and cholesterol [2]. A marked reduction in their measure following flavonoid exposure could correlate to the downregulation in the cholesterol biosynthesis pathway previously discussed and/or indicate a corresponding reduction in the production of uric acid [2]. Uric acid is the product of purine catabolism. In this pathway, the nucleotides adenine or guanine are catabolized in several ways that result in the formation of xanthosine or hypoxanthine [2]. In the last two steps of the process, the rate-limiting enzyme Xanthine Oxidase degrades these products to xanthine and then xanthine to uric acid. When elevated, uric acid levels in circulation increase the chance for precipitation of monosodium urate crystals into joints and tissues. These crystals, with their needle-shaped negative birefringence, are highly inflammatory and destructive in tissues [33,34,35]. They are also central to the presentation of Gout and its resulting arthropathy [33,34,35]. In addition, hyperuricemia, or the elevation of uric acid in the blood, also contributes to the formation of cardiovascular disease, kidney disease, and atherosclerosis [36,37,38,39].

### 5.1. Effects on L-Alanine, Lactate, and the Urea Cycle

In our lab’s past work, the leading metabolites whose downregulation emerged following cellular exposure by chrysin were the inter-related molecules of lactate, L-alanine, and pyruvate [2]. These three molecules can be interconverted for tissue-specific needs. L-alanine, a product of pyruvate and lactate metabolism, can be released by non-hepatic cells and taken up by the liver for use in gluconeogenesis, as a carrier of ammonia for the urea cycle, or as an amino acid substrate for protein synthesis. L-alanine can also be used as an amino acid source of ATP. Metabolomics revealed a statistically significant reduction in L-alanine and Lactate, with a less pronounced reduction in pyruvate [1,2]. From this, a conclusion can be drawn that the mobilization of lactate is not being directed to alanine or its subsequent use for gluconeogenic export or the urea cycle [2]. As these cells are incapable of performing gluconeogenesis or forming urea, it is assumed that the reduction in L-alanine and Lactate signals increased uses of these molecules for protein synthesis, shunting through Pyruvate and Acetyl-CoA into ketogenesis, and/or ATP production. 

Another interesting and centralized molecule in our biochemical pathways is carbamoyl phosphate [2]. It represents the entry point for either the urea cycle or the pathway of pyrimidine synthesis [2]. It is formed from free ammonia and carbon dioxide sources through the conversion of *N*-acetyl glutamate, its precursor, by the enzyme Carbamoyl Phosphate Synthase I (CPSI) or Carbamoyl Phosphate Synthase II (CPSII) [2]. CPSI is found in the mitochondrial matrix and forms carbamoyl phosphate for the urea cycle, while CPSII is found in the cytosol and forms carbamoyl phosphate for pyrimidine synthesis [2]. *N*-acetyl glutamate is formed by the enzyme *N*-acetyl glutamate Synthetase from glutamate and Acetyl-CoA as substrates [2]. When glutamate and *N*-acetyl glutamate are high, CPSI and CPSII enzymes are upregulated [56,57]. Further, Arginine, a major nitrogen-containing intermediate of the urea cycle that builds when the cycle is functioning below needed levels, would signal upregulation of *N*-acetyl glutamate synthase [58,59,60,61,62]. This causes an increase in *N*-acetyl glutamate and, indirectly, the upregulation of CPSI with the purpose of increasing the urea cycle to clear this excess amino acid and its nitrogens [58,59,60,61,62]. In the urea cycle, carbamoyl phosphate is used as a substrate with ornithine to create the first intermediary of the cycle, Citrulline [59,60,61]. Further additions of carbon and nitrogen from amino acid donors ultimately result in the formation of arginine. Arginine is then cleaved to urea as a mechanism of removal of excess nitrogenous waste from the body. Our metabolomic assessment of these molecules indicated a statistically significant decrease in *N*-acetyl glutamate and glutamate but no changes in the intermediaries of the urea cycle [2]. From this, we conclude that a reduction in the pathways of carbamoyl phosphate occurs under the effects of chrysin but that the urea cycle itself is unaffected [59,60,61,63]. In the de novo setting, an error at the beginning of the urea cycle will often present an elevation in alanine levels, as alanine carries ammonia for this cycle’s purpose [59,60,61,63]. As mentioned, our studies showed a demonstratable reduction in alanine levels rather than an increase. Further supporting the argument that no challenges in the urea cycle are observed [2]. 

It is once again important to note that any articulation of the biochemical relationship between Alanine/Pyruvate, the urea cycle, Acetyl-CoA, and gluconeogenesis must account for the cell-specific abilities to perform these cycles [64]. Only hepatocytes can perform gluconeogenesis as well, with some exceptions that kidneys also perform this function [64]. This highlights the importance of assessing metabolic changes following the application of an intervention in the de novo setting. Then, observation of relationships between pathways and respective cellular changes, given their unique functions, would allow more accurate conclusions to be drawn.

### 5.2. Reduction of Pyrimidine Synthesis 

Carbamoyl phosphate, as mentioned, is a molecule involved in both the urea cycle and in pyrimidine synthesis [56,60,61]. Alternatively, nitrogen and carbons may be added to carbamoyl phosphate from glutamine and aspartate amino acids to build a pyrimidine base [2]. Eventually, this pathway will form Orotic Acid, which can finally be placed onto ribose sugar and form the pyrimidine nucleoside orotidine [56,60,61]. Further enzymatic activity can form the pyrimidine nucleotides, starting with Uridine Monophosphate [65], which is a ribose sugar, a pyrimidine base known as uracil, and 5′ phosphate group [56,60,61]. To assess the effects of flavonoids on these pathways, metabolomics was performed, and changes in metabolites were measured. In the presence of chrysin, a reduction in orotidine, the carbamoyl phosphate precursor *N*-acetyl glutamate, as well as end products uracil, cytosine, UMP, and cytosine monophosphate (CMP) were all observed [2]. Measurement of the urea cycle intermediaries L-arginine and D-ornithine demonstrated no changes. With the reduction in *N*-acetyl glutamate, glutamate, and orotidine, the products of this pathway, but no reduction in intermediaries of the urea cycle, it can be concluded that chrysin downregulates the pyrimidine synthesis pathway without affecting the cell’s ability to export ammonia to the urea cycle [2]. 

The pyrimidine synthesis pathway is often the target of treatment for cancers and proliferative disorders. Pyrimidines and purines form the building blocks for nucleic acid synthesis, and thus, DNA replication [2,66]. Proliferations, such as in tumor growth, undergo rapid and frequent cellular division that relies prodigiously on the nucleotides as substrates to perpetuate the cancer’s growth [56,58,62,66]. Starving these proliferative pathologies of the needed material for cellular division is the basis for treatments.

Excess nucleotide synthesis can also cause the excess nucleotides to be incorporated into excess non-coding RNA such as microRNAs [2,67,68]. These types of RNA typically serve to modify the translation of mRNA by either binding to its microRNA Response Element (MRE) with perfect specificity or not. The former causes the degradation of the mRNA [2,67,68]. These modifications to mRNA by microRNA are responsible for the proper and optimal levels of cellular metabolism, cellular proliferation, and apoptosis [67,68]. Despite no findings in our past studies that would correlate to anti-cancer properties of Apigenin, it too has a significant number of publications in the literature stating a complimentary anti-cancer activity [5,69,70,71,72].

### 5.3. Effect on Linoleic Acid Metabolites by Apigenin

a. Adrenic acid-mediated beneficial effects:

Previous works have demonstrated linoleic acid’s potential to reduce cholesterol by lowering LDL and by increasing the conversion of cholesterol to bile and improving vascular health [73,74,75], especially when compared to saturated fatty acids. In 2014, a meta-analysis by Farvid et al. concluded that reducing oral intake of saturated fatty acids with linoleic acid reduced the incidence of cardiovascular disease and coronary artery disease by 9% [76]. An additional meta-analysis in 2019 concluded a similar benefit to cardiovascular health from linoleic acid [77]. Other studies have made contrary claims that linoleic acid is responsible for an increased risk to cardiovascular health [78,79]. Detractors of these contradicting studies note the overly high levels of linoleic acid used for their conclusions. These contradictions likely highlight the importance of maintaining balanced levels of linoleic acid metabolites and the specific roles of each metabolite. The primary metabolite from which linoleic acid’s many biochemical effects stem is arachidonic acid [2]. Assessment of metabolomics demonstrates that apigenin can modulate the metabolism of arachidonic acid. When exposed to Apigenin, fibroblast cells demonstrated unaffected cellular levels of arachidonic acid [2]. Yet statistically significant elevations of its metabolite adrenic acid can be observed. Indicating a shift of arachidonic acid towards adrenic acid from other metabolic mechanisms when cells are exposed to Apigenin. Arachidonic Acid is a 20-carbon metabolite of linoleic acid that is incorporated into the cell’s plasma membrane [2]. It can then be removed from the membrane by the phospholipase A2 enzyme and used in many biochemical pathways. Several of these pathways are related to homeostasis, inflammation, healing, and coagulation through the production of eicosanoids like prostaglandins, thromboxanes, leukotrienes, prostacyclins, and others [2]. The traditional schema related to arachidonic acid metabolism is that this omega-6 fatty acid primarily mediates a pro-inflammatory response [2]. As stated, an alternative fate of arachidonic acid is via further elongation of two additional carbons to the carboxylic end of arachidonic acid to form adrenic acid [2]. Like arachidonic acid, adrenic acid utilizes the cyclo-oxygenase and cytochrome enzymes for metabolism. The two fatty acids compete for the enzyme’s use, and when in elevation, adrenic acid is capable of halting the conversion of arachidonic acid to other metabolites, such as pro-coagulatory prostaglandins. Adrenic acid is found highly in the adrenal gland and endothelium. The metabolites of adrenic acid are like those of arachidonic acid, but in the form of epoxy fatty acids and further to dihomo-eicosanoids [80,81]. Previous studies have demonstrated that the dihomo metabolites of adrenic acid are capable of causing hyperpolarization and vasorelaxation in the coronary vessels and other arteries by activating the K+ channels of the vessel’s smooth muscle [80,81]. A large review of linoleic acid metabolites indicates that the dihomo metabolites are likely those responsible for the anti-inflammatory and anti-coagulation aspects of linoleic acid metabolites’ many properties, antagonize its inflammatory metabolites formations, and may be even more potent than eicosapentaenoic acid (EPA) [82]. Further, metabolites may reduce endoplasmic reticulum stress and inflammatory pain [83]. An in vivo study in 2020 saw a reduction in arthritic pain when mice were exposed to adrenic acid [84]. This study was novel as its in vivo setting challenges the schema of arachidonic acid/omega-6 fatty acids as pro-inflammatory.

b. Adverse effects associated with adrenic acid levels:

Conversely, studies have demonstrated negative impacts on elevated adrenic acid levels. Adrenic acid may cause lipotoxicity in hepatocytes and be significantly increased in non-alcoholic fatty liver disease (NAFLD) [85]. Xylaki et al. showed that the intracellular levels of both arachidonic acid and adrenic acid may play a role in increases in α-synuclein protein aggregations in those with Parkinson’s Disease [65]. When also looking at the pathophysiology of the brain, those with schizophrenia have been found to have decreased adrenic acid and elevated docosahexaenoic acid (DHA) in their plasma membranes [65]. This is an interesting point, given that apigenin has been shown to elevate adrenic acid in cellular models but not DHA, which is discussed further elsewhere [86]. Tardy et al. assessed a variety of long-chain fatty acids’ roles in priming Tissue Factor (Factor III) and, thus, their roles in acting as pro-coagulation mediators in thrombin-stimulated endothelial cells. Their conclusions indicate adrenic acid could induce clot formation and atherosclerosis [87]. Such varying conclusions indicate further understanding of the roles of adrenic acid in the in vivo setting.

## 6. Effects on α-Linolenic Metabolites by Apigenin

On the other hand, α-linolenic acid is the polyunsaturated fatty acid precursor for eicosapentaenoic acid (EPA), docosapentaenoic acid (DPA), and docosahexaenoic acid (DHA). These fatty acids are also incorporated into, and essential for, the cell’s plasma membrane [65,73,82,84,87,88]. These Omega-3 polyunsaturated fatty acids have long been associated with reductions in heart attacks, cardiovascular disease, cognitive decline, strokes, and others, as well as adjunct treatment for hypertriglyceridemia and hypercholesterolemia [89]. The conversion of α-linolenic acid to its metabolites shares the same enzymatic process as that of linoleic acid to arachidonic acid; metabolite levels will frequently favor those with higher exogenous intake of the given essential fatty acid. Because the average diet is far greater in linoleic acid over α-linolenic acid, EPA and DHA are frequently found with far lower concentrations in cells. The metabolites of α-linolenic play roles in reducing inflammation, normal neurologic development, reducing depression, and improving cardiovascular health [73]. The role of EPA and DHA to improve cardiovascular health and reduce the risk of coronary artery disease and congestive heart failure is widely reviewed, researched, and accepted [90,91,92]. Specifically, these fatty acids improve endothelial dysfunction by reducing vasoconstriction and the thrombotic state and inhibit the enzymatic production of pro-inflammatory metabolites of arachidonic acid. These events have an early impact on the development of atherosclerosis. Further metabolites from EPA exist in the form of epoxy fatty acids. These epoxy fatty acids have been widely researched towards the production of anti-inflammatory, antioxidant, endothelial protective, antihypertensive, analgesic, antiapoptotic, and anti-endoplasmic reticular (ER) stress effects [83,93,94]. Recently, two large trials have been undertaken which compared the use of exogenous EPA in the form of the FDA-approved and branded pharmaceutical, “Vascepa”, in those with existing cardiovascular disease and diabetes. These patients presented with hypercholesterolemia and hypertriglyceridemia despite the use of statins. The first clinical trial is entitled—The “Reduction of Cardiovascular Events with Icosapent Ethyl Intervention Trial” (REDUCE-IT) [95,96,97,98,99], and the second is “Effect of Icosapent Ethyl on Progression of Coronary Atherosclerosis in Patients with Elevated Triglycerides on Statin Therapy” (EVAPORATE) [100]. In both studies, the use of exogenous EPA improved outcomes regarding cardiovascular incidences, atherosclerotic build-up in vessels, and other adverse hypercholesterolemic pathophysiology [96,100]. The direct benefits seen on atherosclerosis formation by EPA in these two trials build on previous work in rodent models and human studies with similar findings [101,102]. Based on clinical trial data involving the administration of both EPA and DHA, no major cardiovascular protection has been noted [103]. One of the reasons could be that DHA has been shown to raise LDL-cholesterol levels [104,105]. When reviewing the metabolomics of fibroblast cells after exposure to apigenin and Chrysin, a statistically significant elevation of EPA was observed [2]. Interestingly, small increases in DHA and docosapentaenoic acid were observed, but not to the level of significance [2]. This ability of apigenin to increase EPA without elevating DHA and its long-term elevations in LDL may mark apigenin as an additional source of cardiovascular protection on par with EPA [2], especially in those who have a background or history of hyperlipidemia. Further, a previous review of linoleic acid metabolite effects concluded EPA further acts to amplify the anti-inflammatory metabolites of linoleic acid, which were discussed in the previous section. It should be noted that while EPA may be superior in terms of cardioprotective aspects in hyperlipidemic individuals, DHA is the main fatty acid within the plasma membranes of the brain and eye and is integral for the proper development of these tissues. It is also integral for the proper maintenance of the mitochondrial membrane [73]. At present, not enough studies have been performed comparing the bioavailability of EPA with that of apigenin. This is due in part to the wide variability and non-standardized forms of apigenin. Based on our observation, apigenin may have an EPA-elevating action [2], so it will be important to understand the similarities and differences between apigenin and any exogenous sources of EPA.

## 7. Improved Cellular Availability of EPA by Apigenin

In previous sections, the benefits of apigenin were largely correlated to alterations in EPA levels within the cell following exposure to apigenin. Yet, exogenous EPA is already widely used to exact these benefits. In fact, the National Cholesterol Education Program (NCEP) Adult Treatment Panel III (ATP III) recommends that triglyceride levels be a primary interventional target through the use of FDA-approved purified omega-3 fatty acid esters of EPA and DHA [106]. One such example is the FDA-approved “Vascepa” used and previously discussed in the REDUCE-IT trial. “Vascepa” is unique in that it contains only a high-purity formulation of Icosapent Ethyl [95,96,97,98,100,106]. That is to say, it contains only EPA in its ethyl ester form. In addition, several other FDA-approved medications contain both purified EPA and DHA. They include “Lovaza”, “Omtryg”, and “Epanova”. Vascepa, solely purified EPA, demonstrated better efficacy and greater increases in plasma level EPA within the REDUCE-IT trial than these other EPA and DHA combined forms of exogenous omega-3 fatty acids [95,96,97,98,100,106]. Partly due to only 9.4% of absorbed DHA being retro-converted to EPA, it is understandable that a purely EPA pharmaceutical would provide superior levels of plasma and cellular EPA [107]. A possible limitation of oral supplementation of EPA may be its bioavailability. Oral administration of purified EPA. Bioavailability is a term that captures the complete break-down, absorption, tissue and cellular uptake, metabolism, and excretion of a given substance [107]. When looking at Vascepa, the medication is given as an oral supplement in its Ethyl Ester form [107,108]. Administration as ethyl ester is due to the challenge of maintaining other forms of EPA during the pill’s production. Studying the bioavailability of the various forms of omega-3 fatty acids indicates that ethyl ester is the least bioavailable form. Following ingestion, EPA ethyl esters are de-esterified and absorbed in the small intestine [107]. After absorption, EPA is incorporated into phospholipids, triacylglycerides [85], and cholesterol esters; 99% of the absorbed EPA is incorporated into these molecules [107,108,109]. Only 1% of absorbed EPA is found circulating as free, protein-bound EPA. Of these, free fatty acids, followed by phospholipids, are the most available for cellular and tissue use [107,108,109]. 

Studying the bioavailability and pharmacokinetics of apigenin reveals its bioavailability to be roughly 30%, with other studies indicating between 16–28.6% [107]. Techniques like self-micro emulsifying drug delivery systems, soy-lecithin-based ethosomes, and nanocrystal gel formulations are possible to increase the bioavailability to upwards of 67.39–84.13%. It also has a permeability coefficient (P) of 10^−5^ cm/s, meaning it absorbs well in the intestines but is rapidly metabolized [107,108]. The bioavailability of apigenin is enhanced by its lipophilicity. This hydrophobicity, as well as a slightly acidic nature, allows for its absorption throughout the small intestines. Its greatest absorption rates appear to be in the duodenum [110]. Lipinski’s “Rule of Five” states a potential pharmacologic agent candidate for oral interventions should not have an Octanol-water partition coefficient (logP) > 5 [72]. Meaning the drug cannot be too lipophilic [72,110]. Apigenin has a logP of 2.85, which indicates it is lipophilic enough to be absorbed in the intestine, can be taken up by many tissues, and crosses the blood-brain barrier, but not too lipophilic [72,111]. In addition, apigenin has a slow clearance from plasma and liver compared to other flavones [72,107,110]. Maximum serum concentration is reached within 0.5–2.5 h, and its half-life is 2.5 h (plus or minus 0.5 h). Apigenin is considered unfeasible for reaching therapeutic levels through oral consumption via food sources containing naturally occurring apigenin [72,107,110]. This marks the great importance of further studies that could lead to a pure Apigenin capsular oral therapy [72,107,110]. Intravenous administration of apigenin also easily reaches therapeutic levels [72,107,110,111]. When considering the advantages of purified EPA administration compared to apigenin, no study directly compares these molecules. Further work on this matter will be important.

### 7.1. Alteration of Cellular Metabolism

Based on our previous works, chrysin demonstrates robust anti-cancer properties through modulation of pyrimidine synthesis [2,3]. Apigenin, on the other hand, emerges as a stronger cardioprotective flavonoid. Specifically, linoleic acid and α-linolenic acid, omega-6, and omega-3 PUFAs, respectively. They are essential within the diet as the human body is not able to desaturate or produce a double bond beyond carbon 9 in a fatty acid chain [2,3]. Since 1929, it has been accepted that these two polyunsaturated fatty acids (PUFAs) are necessary for maintaining vascular and circulatory health [88]. In our previous study assessing the metabolite changes in cellular culture, modulation of the metabolism of linoleic acid and α-linolenic acid was observed to have statistical significance, as well as the primary metabolic pathway affected by apigenin [2,3]. No appreciable change when cells were exposed to chrysin was observed in these pathways [2,3]. The anti-inflammatory and vascular protective effects of apigenin through its action on PUFAs are coupled with the reductions in uric acid and cholesterol, as seen with both apigenin and chrysin [88]. Elevations in either cholesterol or uric acid would themselves contribute to inflammatory and vascular pathology processes. Apigenin and chrysin could be ideal agents to reduce systemic cholesterol and uric acid excess and bring about cellular metabolic homeostasis.

### 7.2. Limitations and Other Directions

Most parts of our review were just focused on the impact of apigenin and chrysin on cellular effects regulating cardiovascular health, as per our lab findings and other findings that support our work. Rather than writing a generalized review, we were focused on highlighting the pharmacological properties of these two closely related flavonoids on metabolic health. Majority of the cited work have not studied in depth the pharmacokinetic properties of these two flavonoid compounds especially the ones involving in vivo studies which are a major void in the area of flavonoid research especially when the focus is on systemic health. Further studies involving apigenin and chrysin with pharmacokinetic properties, including bioavailability studies, are highly warranted. Before concluding our work, we would also like to shed some light on other directions where both apigenin and chrysin can demonstrate very similar pharmacological properties and bring about similar systemic benefits, which are described in Table 2. Our is the only work that has detailed, in-depth information regarding their overlapping and also diverging cellular mechanisms, which is unique and novel. These findings were from the PubMed database, including keywords like apigenin, chrysin, systemic effects, and pharmacological properties.

## 8. Conclusions

Past works by our laboratory assessed the effects of the flavonoids apigenin and chrysin through transcriptome analysis and widespread metabolomics. The results of these studies indicate a modulating effect by these two phytochemicals that downregulate biosynthetic pathways and gluconeogenesis with a divergence towards ketogenesis. The strongest effect of both chrysin and apigenin seems to be on the biosynthesis of Cholesterol. Transcription factors from virtually every gene coding for the enzymes of this pathway were decreased by both chrysin and apigenin. From this, apigenin and chrysin may represent a potential avenue of treatment related to hypercholesteremia that is worthy of exploration. 

## Figures and Tables

**Table 1:** Summary of Cellular Effects through Transcriptome and Wide-Spread Metabolomics Changes Following Exposure to Apigenin and Chrysin.

Predominant Flavonoid Producing the Observed Change	Metabolomic or Transcriptome Change Observed with Statistical Significance	Conclusions
Apigenin and Chrysin	Reduced RNA transcripts of gene Hmgcs1, which codes for the enzyme HMG-CoA Synthase	Downregulation of cholesterol biosynthesis [2,3]
	Reduced RNA transcripts of gene Mvk, which codes for the enzyme Mevalonate Kinase	Downregulation of cholesterol biosynthesis [2,3]
	Reduce RNA transcripts of gene Pmvk, which codes for the enzyme phosphomevalonate kinase	Downregulation of cholesterol biosynthesis [2,3]
	Reduced RNA transcripts of the gene Idil, which codes for the enzyme Isopentyl-Diphosphate Delta Isomerase 1	Downregulation of cholesterol biosynthesis [2,3]
	Reduced RNA transcripts of the gene Fdps, which codes for the enzyme Farnesyl Diphosphate Synthase	Downregulation of cholesterol biosynthesis [2,3]
	Reduce RNA transcripts of the gene Msmo1, which codes for the enzyme Methylsterol Monooxygenase 1	Downregulation of cholesterol biosynthesis [2,3]
	Reduced RNA transcripts of the gene Lss, which codes for the enzyme Lanosterol Synthase	Downregulation of cholesterol biosynthesis [2,3]
	Reduced RNA Transcripts of LDLR, which codes for the Low-density lipoprotein receptor	Alterations in cholesterol homeostasis [2,3]
	Reduced RNA transcripts of the gene ELOVL6, which codes for Fatty Acid Elongase enzymes	Downregulation of fatty acid biosynthesis [2,3]
	Reduced cellular levels of the ER-bound protein, SREBP’s transcription factors Srebf1 and Srebf2	Downregulation of fatty acid synthesis and cholesterol biosynthesis [2,3]
	Reduced RNA transcripts of the gene Fasn, which codes for the enzyme Fatty Acid Synthase	Downregulation of fatty acid biosynthesis [2,3]
	Increased RNA transcripts of the gene Bdh1, which codes for the gene 3-Hydroxybutyrate Dehydrogenase 1	Increased ketogenesis activity [2,3]
	Increased RNA transcripts of the gene Slc16a6, which codes for the Monocarboxylate Transporter 6 (MCT6)	Increased ketogenesis activity [2,3]
	Reduced intracellular 7-dehydrocholesterol levels	Reduced cholesterol biosynthesis and/or uric acid production [2,3]
	Reduced intracellular Xanthosine levels	Reduced cholesterol biosynthesis and/or uric acid formation [2,3]
Chrysin Only	Reduced RNA transcripts of the gene Acaca, which codes for the Enzyme Acetyl-CoA Carboxylase. The rate-limiting step in fatty acid synthesis	Reduced Fatty Acid Biosynthesis [2,3]
	Reduced intracellular L-alanine levels	Reduced gluconeogenesis activity or increased ATP production [2,3]
	Reduced Intracellular levels of Lactic Acid	Utilization of Lactate for fuel without causing gluconeogenesis via L-Alanine [2,3]
	Reduction in intracellular glutamate levels	Decreased utilization of Acetyl-CoA in the pyrimidine synthesis pathway [2,3]
	Reduction in intracellular *N*-acetyl glutamate	Decreased utilization of pyrimidine synthesis pathway [2,3]
	Reduction in intracellular Orotidine levels	Reduce pyrimidine synthesis [2,3]
	Reduced intracellular levels of uracil, UMP, and CMP	Reduce pyrimidine synthesis [2,3]
Apigenin Only	Reduced RNA transcripts of the gene Hmgcr, which codes for the rate-limiting step of cholesterol biosynthesis HMG-CoA Reductase	Downregulation of cholesterol biosynthesis [2,3]
	Increased intracellular levels of the PUFA Eicosapentaenoic Acid (EPA) without statistically significant elevations in docosahexaenoic acid (DHA)	Cardioprotective, anti-oxidant, anti-inflammatory [2,3]
	Elevations in Adrenic Acid and alternate metabolite of Arachidonic Acid	Vasoprotection, endothelial oxidative stress reduction, and divergence from arachidonic Acid inflammatory pathway [2,3].

**Table 2:** **Systemic benefits of apigenin and chrysin**.

Apigenin and Chrysin	Pharmacological Property	Systemic Effects
1	Antioxidant property	Combat oxidative stress [112,113]
2	Anticancer property	Prevent tumor cell growth [114,115]
3	Anti-ischemic property	Preventing cardio and cerebrovascular ischemia [116,117]
4	Anti-diabetic property	Prevent hyperglycemia and associated complications [118,119]
5	Inhibition of neurodegeneration	Attenuates neurodegenerative diseases [120,121]
6	Anti-inflammatory property	Prevents systemic and organ-specific inflammation [122,123]
7	Anti-infective property	Demonstrate antibacterial and antiviral effects [124,125]
